# Parvovirus B19V DNA contamination in Chinese plasma and plasma derivatives

**DOI:** 10.1186/1479-5876-10-194

**Published:** 2012-09-17

**Authors:** Wei Zhang, Ling Ke, Li Changqing, Yan Zhang, Wuping Li

**Affiliations:** 1 Institute of Blood Transfusion, Chinese Academy of Medical Sciences and Peking Union Medical College, Hua Cai Road 26 Hao, Dong San Huan Road Er Duan, Chengdu, Sichuang, 610052, China; 2 Cheng Du Rongsheng Pharmaceuticals Co. LTD, Hua Cai Road 26 Hao, Dong San Huan Road Er Duan, Chengdu, Sichuang, 610052, China; 3 Institute of Pathogen Biology, Chinese Academy of Medical Sciences and Peking Union Medical College, Beijing, China

## Abstract

**Background:**

To ensure the safety of plasma derivatives, screening for human parvovirus B19V genomic DNA in donated plasma using a pooling strategy is performed in some countries. We investigated the prevalence of B19V DNA and anti-B19V antibodies in Chinese plasma pools, plasma derivatives and plasma donations to evaluate the risk posed by B19V.

**Methods:**

Using a Q-PCR assay developed in-house, we tested for B19V genomic DNA in 142 plasma pools collected between January 2009 and June 2011 from two Chinese blood products manufacturers. Plasma derivatives collected between 1993–1995 (10 batches of albumin, 155 batches of intravenous immunoglobulin, IVIG) and 2009–2011 (50 batches of albumin, 54 batches of IVIG, 35 batches of factor VIII, 7 batches of fibrinogen, and 17 batches of prothrombin complex concentrate, PCC) were also tested for B19V contamination. In addition, B19V genome prevalence in minipools(including 90 individual donations) of 49680 individual plasma samples collected between August 2011 and March 2012 by a single Chinese manufacturer was investigated. IgM/IgG was also investigated in plasma pools/derivatives and in minipools with B19V-DNA titers above 1x10^4^ and 1x10^6^ geq/mL using B19 ELISA IgM/IgG assay(Virion-Serion, Würzburg, Germany), respectively.

**Results:**

B19V-DNA was detected in 54.2% of plasma pools from two Chinese blood product manufacturers; among recently produced blood products, B19V was detected in 21/54 IVIG samples, 19/35 factor VIII samples, 6/7 fibrinogen samples, and 12/17 PCC samples, but not in albumin samples. The levels of B19V-DNA in these samples varied from 10^2^-10^7^ geq/mL. In samples with >10^4^ geq/mL genome DNA, B19V-specific IgG was also found in all corresponding plasma pools and IVIG, whereas none was detected in the majority of other plasma derivatives. Screening of plasma donations indicated that most minipools were contaminated with B19V-DNA (10^2^-10^8^ geq/mL) and one donation had 1.09 × 10^10^ geq/mL B19V genomic DNA along with a non-classical IgG/IgM profile.

**Conclusions:**

Despite the implementation of some inactivation/removal methods designed to prevent viral contamination, B19V DNA was detectable in Chinese plasma pools and plasma derivatives. Thus, the introduction of B19V screening and discard donation with high viramic concentration for Chinese plasma donors would be desirable.

## Background

Human parvovirus B19V is a small icosahedral, non-enveloped single-stranded DNA viral pathogen that can cause a variety of diseases, including erythema infectiosum (fifth disease), arthritis, transient aplastic crisis, chronic anemia (in immunocompromised patients), hydrops fetalis, and fetal death [1-4]. The main route of B19V transmission is via the respiratory route, although it can also be transmitted vertically and via blood transfusion and organ transplantation [5]. B19V infection usually happens during childhood; however, 40–60% of adults are still susceptible to primary infection [6,7]. Depending on assay sensitivity and epidemic incidence, the prevalence of B19V DNA in blood donors can be up to 1%, with virus titers reaching 1 × 10^14^ geq/mL during early acute infection, although affected individuals are often asymptomatic. This level of prevalence is sufficient to contaminate most plasma pools used for fractionation [8,9], and, eventually, plasma derivatives that are usually prepared from pools of several thousand donations. One study demonstrated that, overall, 85% (60–100% depending on manufacturer) of plasma pools, 25% of albumin samples, 100% of factor VIII, 20% of IVIG, and 75% of intramuscular immunoglobulin preparations contained B19V DNA [10]. Viral load in those samples ranged from 1 × 10^2^ to 1 × 10^6^ geq/mL. Another study reported a high prevalence (over 60%) of B19V DNA in factor IX, factor VIII, PCCs, and plasma pools with viral loads of 1 × 10^2^ to 1 × 10^8^ geq/mL [11].

The small size (20–25 nm in diameter) and non-enveloped nature of B19V render it difficult to remove by filtration methods and very resistant to many virus inactivation procedures used in the production of plasma derivatives, including solvent/detergent (S/D) and heat treatment. The transmission of B19V through the administration of S/D-treated [12] and certain dry heat-treated blood products has already been documented [13-15]. B19V can also be transmitted by blood component [16,17], while one study indicated only high concentration containing component can cause infection [18]. Transmission of B19V by blood and blood products and its resistance to common viral inactivation/removal methods raises the importance of detecting B19V prior to blood transfusion. The FDA has proposed a limit of 10^4^ geq/mL for manufacturing pools destined for all plasma derivatives to reduce the potential risk of transmission [19,20]. Similarly, European Pharmacopoeia has imposed a limit of 10^4^ IU/mL for levels of B19V in anti-D immunoglobulins and pooled virus inactivated plasma.

Many studies have demonstrated the presence of B19V DNA in plasma pools and plasma-derived products [11,21-24]; however, the prevalence of B19V DNA in Chinese blood products and plasma pools has not been extensively investigated. In this study, we aimed to determine the frequency and level of B19V DNA contamination in plasma pools collected during 2009–2011, and in plasma-derived products produced during two periods,1993–1995 (albumin, IVIG) and 2009–2011(albumin, IVIG, factor VIII, Fibrinogen), under license in China. Since no B19V Nucleic Acid Testing (NAT) regimen has previously been proposed for Chinese blood products manufacturers, the results of the present study will hopefully provide a significant advance in this area and have a positive impact on policy development with regard to blood safety in China.

## Materials and methods

### Samples

We tested 142 industrial pools used for fractionation into plasma derivatives between January 2009 and June 2011 from two regional different Chinese blood products manufacturers. The study also included 10 batches of albumin and 155 batches of IVIG prepared between 1993 and 1995 and stored in our lab, and 50 batches of albumin, 54 batches of IVIG, 35 batches of factor VIII, 7 batches of fibrinogen, and 17 batches of PCC prepared between 2009 and 2011. We also investigated B19V genome DNA contamination in minipools of 49680 individual plasma samples collected between August 2011 and March 2012 by one of those two Chinese manufacturers. A protocol to investigate B19V DNA in plasmapheresis donors was developed. Briefly, aliquots of individual plasma samples (10 μL) from 90 human donations were pooled, and orerall 552 mini-pools were obtained. DNA was extracted from a 200 μL mini-pool as described above; 5 μL of extracted DNA was used for PCR amplification and quantitation of B19V DNA. When a test pool exceeded the threshold of 10^6^ geq mL B19V DNA, the pool was broken down via subpools of 10 donations, and all individual donations in a reactive subpool were tested to identify the highly viraemic donation.

### B19V DNA extraction and quantitation by Q-PCR

A volume of 200 μL pooled source plasma or plasma derivative was subjected to nucleic acid extraction using the TIANamp virus DNA/RNA kit (TIANGEN), according to the manufacturer’s instructions. All DNA extracts were stored at -80°C prior to PCR analysis. IUs were adjusted to genome equivalent (geq) using plasmid pYT-103, which contains the sequence of the B19V Genotype 1 genome. The conversion ratio from geq to IU is 1.02. Before quantitation of B19V genome of the DNA extracts, the specificity of the primers and the sensitivity of the detection system were tested as previously described [25]. The DNA extracted from the WHO International standards (NIBSC 09/110; 106 IU of B19V DNA/mL) were used to determine the conversion ratio from geq to IU and to validate the specificity of the primers. Meanwhile, a serial dilution of plasmid pYT-103 which contains sequence of B19V Genotype 1 genome was used to investigate the sensitivity of the detection system. Briefly, a serial dilution (10×) of pYT-103 from 5 to 5 × 10^7^ geq per reaction(20 μl) for NAT based assays were tested for the lowest detection limit. If a signal of the lowest standard was detected at more than 40 cycles, the result was interpreted as invalid and repeated the experiment. This warrant the sensitivity of the experiment is much lower than 125 geq/mL and guarantees our data to fulfil the criteria. The sequence of the forward primer is 5^′^-TGCAGATGCCCTCCACCCA-3^′^ and the sequence of reverse primer is 5^′^-GCTGCTTTCACTGAGTTCTTC-3^′^ which are located in the NS1 gene (NS1-PCR) and can amplify all three B19V genotypes, resulting in a 216 bp fragment. Quantitative PCR (Q-PCR) assays were performed on an ABI Prism 7900 Sequence Detection System platform (Applied Biosystems, Foster City, CA) using a SYBR Green I (Molecular Probes, Eugene, OR) PCR mix. Briefly, the 20 μL reaction mix consisted of 10 μL FastStart Universal SYBR Green Master (Rox) (Roche Diagnostics, Indianapolis, IN, USA), 12.5 pmol of each primer, and 5 μL extract DNA as a template. Hot-start amplification was performed under the following conditions: 1 cycle of 95°C for 10 min; 45 cycles of 95°C for 15 s, 60°C for 30 s, and 72°C for 30 s; and a final cycle of 95°C for 15 s, 60°C for 15 s, and then gradually increased to 95°C in 30 min at a ramp rate of 2%. Detailed Q-PCR procedures have been previously described [26]. Q-PCR detected B19V DNA positive samples were confirmed by nested PCR (nPCR) using the conserved primers located in the NS1 region described previously [25].

### B19V serologic assays

Testing for B19V-specific antibodies was performed with a commercial assay kit (parvovirus B19V immunoglobulin IgG or IgM enzyme immunoassay, Virion-Serion, Würzburg, Germany) according to the manufacturer’s recommendations. The detecting kit of IgG was against B19V recombinant VP1 and the detecting kit of IgM was against recombinant VP1/VP2. All the plasma products and plasma pools with B19V genomic DNA titers higher than 10^4^ geq mL and resolved viraemic individual donations were tested. Samples were initially tested a single time; if results were equivocal, the assay was repeated and an unambiguous result was taken as the final value for the specimen.

## Results

### Q-PCR sensitivity and specificty

To determine the sensitivity of the Q-PCR assay, 10 individual plasma samples with no detectable anti-B19V specific IgM or IgG were selected as negative controls. When tested by Q-PCR, the 10 negative samples demonstrated no specific amplification after a total of 50 PCR cycles. The detection limit of the B19V DNA Q-PCR assay was determined by performing 10-fold serial dilutions of plasmid PYT-103 into plasma negative for B19V genomic DNA. The highest dilution at which all four replicates were positive was taken as the end-point. The detection limit was 5 geq of B19V DNA in each PCR reaction, which is equivalent to 125 geq/mL per sample. As our previously study indicated [25], our in-house Q-PCR was efficient in amplifying all three B19V genotype. For screening, reactive plasma pool samples were re-tested in duplicate and confirmed by nested PCR (data not shown). If a signal of the lowest standard was detected at more than 40 cycles, the result was interpreted as invalid and repeated the experiment.

### Prevalence of B19V DNA and antibody in plasma pools

To investigate the prevalence and levels of B19V DNA in plasma pools, NAT was performed using pools destined for plasma derivatives made by two manufacturers of Chinese blood products in the period 2009–2011. As shown in Table 1, 24 of 80 (30%) plasma pools from company A were positive for B19V DNA. Of these 24 pools, seven contained >10^4^, and seventeen < 10^4^ geq/mL. However, in the same period, plasma pools from company B had a much higher prevalence of B19V genome DNA; 53 of 62 (85.5%) plasma pools were positive for B19V DNA, among which 26 contained more than 10^4^ geq/mL and 27 less than 10^4^ geq/mL. The quantity of B19V DNA varied from 10^2^ to 3.07 × 10^8^ geq/mL. Plasma pools with a viral load higher than 10^4^ geq/mL are shown in Table 2. The anti-B19V IgG and IgM titers were also determined in plasma pools that contained levels of B19V DNA higher than 10^4^ geq/mL (Table 2). All plasma pools were positive for IgG, with titers in the range of 6–52.5 IU/mL. Twenty one of 33 (65.6%) batches of plasma pools contained IgM, and the titer varied from 5.4–50 IU/mL. There was no correlation between levels of B19V DNA content and the titer of IgG/IgM.

**Table 1:** The prevalence and levels of B19V DNA in plasma pools for fractionation from two blood product manufacturers

**Manufacturer**	**Positive lots/lots tested (%)**	**B19V DNA (geqmL) among positive lots**			
		**≥ 10**^**4**^	**≥ 10**^**3**^	**≥ 10**^**2**^	**Log geq ± SEM**
Company A	24/80 (30%)	7	9	8	3.96 ± 1.67
Company B	53/62 (85.5%)	26	26	1	4.79 ± 1.77
Total	77/142 (54.2%)	33	35	9	4.95 ± 1.67

*SEM* standard error of log geomean.

**Table 2:** **B19V DNA and antibodies in plasma pools with B19V genome titers >10**^**4**^ **geq/mL**

**Sample source**	**Company/Lot number**	**Viral load (geq/mL)**	**IgG (IU/mL)**	**IgM (IU/mL)**
Company A	A-1	1.17 × 10^8^	15.2	Ne
	A-2	9.43 × 10^7^	15.5	6.8
	A-3	2.13 × 10^6^	17.5	Ne
	A-4	1.59 × 10^6^	12.9	Ne
	A-5	1.63 × 10^5^	21	12.2
	A-6	9.42 × 10^4^	17	16.5
	A-7	1.42 × 10^4^	17.9	9.3
Company B	B-1	3.07 × 10^8^	14.5	50
	B-2	2.63 × 10^8^	20	21.5
	B-3	1.83 × 10^8^	22	Ne
	B-4	1.44 × 10^8^	30	Ne
	B-5	1.28 × 10^8^	24.8	Ne
	B-6	9.88 × 10^7^	10.2	18.8
	B-7	9.22 × 10^7^	16	5.4
	B-8	2.79 × 10^7^	21	31.8
	B-9	1.29 × 10^7^	9.3	8.2
	B-10	8.78 × 10^6^	39	Ne
	B-11	2.1 × 10^6^	20.5	10.5
	B-12	1.02 × 10^6^	45	42
	B-13	8.41 × 10^5^	11.7	6.4
	B-14	5.9 × 10^5^	11.8	13
	B-15	5.23 × 10^5^	12.5	12.5
	B-16	3.59 × 10^5^	18.8	25
	B-17	2.72 × 10^5^	6	Ne
	B-18	8.22 × 10^4^	7.5	14.8
	B-19	5.16 × 10^4^	24.5	25.5
	B-20	3.86 × 10^4^	52.5	Ne
	B-21	1.99 × 10^4^	28	6
	B-22	1.86 × 10^4^	23.5	Ne
	B-23	1.55 × 10^4^	14.9	21
	B-24	1.49 × 10^4^	21.6	14.1
	B-25	1.34 × 10^4^	24.8	Ne
	B-26	1.07 × 10^4^	11.4	Ne

### B19V DNA and antibodies in plasmapheresis donors

A total of 49680 individual plasma samples collected from company A between August 2011 and March 2012 were pooled into 552 pools(each pool contained 90 individual donation) and tested for B19V DNA contamination. Of these, one pool contained B19V DNA at a level higher than 10^8^ geq/mL; 24, 64, 342 and 105 contained B19V DNA at levels between 1–10 × 10^5^ geq/mL, 1–10 × 10^4^] geq/mL, 1–10 × 10^3^ geq/mL, and 1–10 × 10^2^ geq/mL, respectively; and 16 pools had no detectable B19V DNA. Overall, 97% (536 of 552) mini-pools were contaminated with B19V DNA, however, most of the pools contained low B19V genome DNA. The pool containing >10^6^ geq/mL was resolved to identify an individual highly viraemic donation with 1.09 × 10^10^ geq/mL B19V genomic DNA. IgM and IgG were negative at the index time. The results of retrospective and follow-up tests are shown in Table 3. B19V DNA titers were lower at subsequent donations and the virus titer abruptly decreased to 2.07 × 10^4^ geq/mL 10 weeks after the index time. Surprisingly, the retrospective sample, collected two weeks before the index time, was IgG positive with 17.4 IU/mL and had a B19V DNA titer of 2.29 × 10^6^ geq/mL. IgM and IgG became detectable two weeks after the index time. Although samples from the following four donations remained positive for both antibodies, the IgG level continued to increase, whereas the IgM level decreased.

**Table 3:** B19V DNA and anti-B19V antibodies in retrospective and follow-up specimens from an individual highly viraemic donation

**Sampling date**	**Viral load (geq/mL)**	**IgG (IU/mL)**	**IgM (IU/mL)**
2011.11.25	2.29 × 10^6^	17.4	Ne
**2011.12.08**	**1.09 × 10**^**10**^	**Ne**	**Ne**
2011.12.22	6.26 × 10^6^	34.2	24.5
2012.1.5	1.68 × 10^5^	69	12
2012.1.19	1.60 × 10^5^	87	8
2012.2.2	5.76 × 10^4^	88.5	7.3
2012.2.16	2.07 × 10^4^	75	6

Ne Negative. Bold text indicates the index sample.

### B19V DNA in plasma derivatives

Table 4 summarizes the B19V DNA content in plasma products. Overall, 71/328 (21.6%) of these products were contaminated with B19V DNA. In 3 of 10 batches of albumin produced during 1993 to 1995, B19V DNA was detectable; however, the products contained <10^4^ geq/mL. B19V DNA was not detected in any of the 50 batches of albumin produced during the period 2009–2011. For IVIG, 6.5% (10 of 155) of tested batches produced from 1993 to 1995 contained B19V DNA at concentrations up to 7.75 × 10^6^ geq/mL, eight batches were highly contaminated (≥10^4^ geq/mL) and two contained < 10^4^ geq/mL. The ratio of B19V DNA positive IVIG produced during 2009–2011 was 38.9% (21 of 54 batches), which was significantly higher than that produced during 1993 to 1995. However, 18 positive batches contained B19V DNA at <10^4^ geq/mL, and only three batches were highly contaminated (≥10^4^ geq/mL). Fifty four percent (19 of 35) of factor VII samples tested positive for B19V DNA with levels of up to 1.87 × 10^5^ geq/mL, among which six batches were highly contaminated (≥10^4^ geq/mL) and 13 batches moderately contaminated (<10^4^ geq/mL). Among seven batches of fibrinogen produced between 2009 and 2011, six (85.7%) were contaminated with B19V genomic DNA, three were highly contaminated (≥10^4^ geq/mL), and three contained less than 10^4^ geq/mL. For PCC produced in the same period, 53% (12 of 17 batches) were contaminated, with four and eight containing B19V DNA levels higher and lower, respectively, than 10^4^ geq/mL. B19V DNA and antibodies in plasma derivatives with genome titers >10^4^ geq/mL are shown in Table 5. Among these plasma derivatives, all IVIG were positive for IgG, with titers in the range of 7.5-144 IU/mL, all the other derivatives contained no IgG except two batches Fibrinogen contained low level IgG. For IgM, most of the derivatives were negative, except 3 of 4 batches of PCC and 2 of 3 batches of Fibrinogen contained low level. Besides, two batches of IVIG were positive for IgM with one in low level and one in high level. There was no association of levels of B19V DNA content and the titer of IgM/IgG.

**Table 4:** Prevalence and levels of B19V DNA in plasma derivatives

** Time period**	**Product**	**Positive lots/lots tested(%)**	**B19V DNA (geq/mL)among positive lots**			
			**≥ 10**^**4**^	**≥ 10**^**3**^	**≥ 10**^**2**^	**Log geq ± SEM**
1993–1995	albumin	3/10 (30%)	0	2	1	3.24 ± 0.44
	IVIG	10/155 (6.5%)	8	2	0	4.92 ± 1.13
2009–2011	albumin	0/50 (0)	0	0	0	/
	IVIG	21/54 (38.9%)	3	3	15	2.98 ± 0.65
	factor VIII	19/35 (54.3%)	6	9	4	3.76 ± 0.94
	Fibrinogen	6/7 (85.7%)	3	3	0	4.35 ± 1.26
	PCC	12/17 (53.0%)	4	4	4	3.54 ± 1.13
Total		71/328 (21.6%)	24	23	24	

*SEM* standard error of log geomean.

**Table 5:** **B19V DNA and antibodies in plasma derivatives with genome titers >10**^**4**^**geq/mL**

** Sample**	**Lot number**	**Viral load (geq/mL)**	**IgG (IU/mL)**	**IgM (IU/mL)**
Fibrinogen	1	6.64 × 10^4^	Ne	Ne
	2	6.29 × 10^6^	13	11.2
	3	1.05 × 10^4^	13	12
Factor VIII	1	1.22 × 10^5^	Ne	Ne
	2	1.35 × 10^5^	Ne	8.5
	3	2.31 × 10^4^	NA	NA
	4	3.08 × 10^4^	NA	NA
	5	1.87 × 10^5^	NA	NA
	6	1.17 × 10^5^	Ne	Ne
IVIG	1	1.19 × 10^4^	144	Ne
	2	1.09 × 10^4^	72	Ne
	3	1.22 × 10^4^	138	Ne
	4^§^	1.20 × 10^5^	31	Ne
	5^§^	1.55 × 10^7^	7.5	11.5
	6^§^	2.98 × 10^4^	50	Ne
	7^§^	1.24 × 10^6^	74.9	Ne
	8^§^	2.59 × 10^4^	82	Ne
	9^§^	4.82 × 10^4^	38	Ne
	10^§^	3.14 × 10^4^	95	Ne
	11^§^	3.37 × 10^5^	110	100
PCC	1	3.47 × 10^4^	Ne	Ne
	2	1.05 × 10^5^	Ne	11.5
	3	3.00 × 10^5^	Ne	13
	4	2.05 × 10^4^	Ne	11.2

^§^ Samples collected in the period 1993–1995; Ne, negative; NA, no data.

## Discussion

Blood products have been widely used for the prevention and treatment of a variety of life-threatening injuries and diseases; however, the contamination of these products with viruses also poses a great threat to patients. Due to the implementation of a variety of measures such as donor selection, virus inactivation/removal, and the testing of donations and of plasma pools, the risk of transmission of blood-borne viruses, especially hepatitis B virus (HBV), hepatitis C virus (HCV) and human immunodeficiency virus (HIV), through plasma and plasma products has been dramatically reduced in developed countries. In 2002, a guideline (the guideline of the technological methods and validation of viral removal/inactivation of blood products, No. 2002–160) was implemented in China with the aim of improving the virus safety of plasma derivatives. This guideline sets the Chinese standard for performing virus validation studies on medicinal products derived from human plasma. The removal/inactivation of viruses in these products requires the implementation of production procedures including S/D treatment, heat (pasteurization and dry heating), and filtration [27]. For coagulation factor concentrates and immunoglobulin, one or several combinational methods should be used to inactivate/remove enveloped and non-enveloped viruses, and albumin manufactured by cold ethanol fractionation must be heat-treated (pasteurized) in the final container. These methods are very effective for the inactivation/removal of the aforementioned principal viruses (HIV, HBV, and HCV); however, non-enveloped viruses, such as B19V, cannot be efficiently removed by these methods and therefore pose a residual risk. This is because (1) plasma pools are prepared from a large number of donations (more than 5000), and it is likely that a high proportion of pools are contaminated with B19V, potentially including some highly contaminated donations (up to 10^14^ geq/mL), and (2) B19V is extremely heat resistant and is small in size, which makes it difficult to remove or inactivate by heat treatment or filtration. B19V DNA contamination of plasma products has been reported in several studies [28-32]; however, the risk posed by Chinese plasma derivatives has not previously been addressed. In this study, coagulation factor concentrates, such as plasma-derived factor VIII, fibrinogen and PCC were found to be highly contaminated with B19V DNA at levels of 10^2^ to 10^7^ geq/mL. IVIG and albumin were moderately contaminated with low levels of B19V DNA.

Parvovirus B19V infection is relatively common worldwide. In immunocompetent individuals, the infection generally occurs without any serious consequences. However, in specific groups, such as pregnant women, patients with underlying hematological problems and patients with immunodeficiency, B19V infections can result in serious complications [33-35]. It is estimated that 50% of all 15-year-olds in the western world have experienced an infection [8]. However, in the elderly population, higher percentages, as high as 80 or 100%, have been observed [36].

A study in our laboratory on blood donors from many Chinese regions reported the NAT testing of samples from 3957 donors using an in-house developed Q-PCR assay for the initial testing and a nested PCR assay for confirmation. The study indicated a 0.58% genome DNA prevalence rate among the blood donor population [26], but the study was not large enough to define range of viral titers, as samples with very high titers are relatively rare. In this study, we investigated B19V DNA contamination in 142 plasma pools from two Chinese manufacturers. As indicated in Table 1, 77of 142 industrial plasma pools (54.2%) were positive and 33 contained B19V-DNA titers higher than 10^4^ geq/mL. In the case of manufacturer A, 30% of plasma pools were positive, but in the case of manufacturer B, the majority (85.5%) were positive. This may reflect a regional difference in B19V infection prevalence. It is very interesting to found the prevalence of B19V in the mini-pool (each contain 90 donations) from a total of 49,680 plasma donations from manufacture A was 97%, much higher than that of other pools collected from both companies during 2009–2011. However, these individual plasmas were collected between August 2011 and March 2012 from company A which located in the south of China. Winter and spring are the epidemic periods of respiratory virus in this area. This may reflect a temporal difference in B19V infection. The quantity of B19V DNA varied from 10^2^ to 3.07 × 10^8^ geq/mL and approximately 43%(33 of 77 positive pools) of pools contained B19V DNA at titers higher than 10^4^ geq/mL, which was sufficiently large to contaminate the products produced from these plasma pools. These data are consistent with previous reports indicating that B19V-DNA is detectable in more than 60% of pools used for the production of plasma products, although usually at relatively low levels [28,37].

In this study, we identified one donation with a B19V DNA titer of 1.09 × 10^10^ geq/mL, which was IgG/IgM negative at the index time. Unexpectedly, B19V DNA and IgG were positive, although the levels were lower than that in a sample collected from the same individual two weeks previously (Table 3). This is not consistent with an acute B19V infection and probably indicates that the donor had a persistent B19V infection, which is usually associated with anomalies in immune status or an immune response that produces B19V antibodies that are ineffective in neutralizing the virus, this result support the data of a previously study [25]. It is likely that, in the days prior to the index time, B19V was reactivated, which would have led to viral replication and a rapid increase in viral load, and, thereby, neutralization of the existing level of IgG. As indicated in Table 3, during the following two months, B19V-DNA decreased rapidly to 2.07 × 10^4^ geq/mL, IgG gradually increased to a plateau, and IgM decreased to a barely detectable level. Other high titer donations are likely to have contributed to a higher prevalence of B19V DNA among plasma pools from manufacturer B. However, unfortunately, samples permitting further investigation were unavailable.

There are several limitations to this study. Firstly, the investigation was confined to the B19V genome content of Chinese plasma pools and plasma derivatives, and the infectivity of the virus in these samples was not determined. Nevertheless, although some inactivation/removal methods have been implemented, these data support the finding that B19V can be transmitted by blood products [38-40] and that the B19V-DNA content reflects, at least partly, contamination with the infectious virus. Secondly, there are about 24 blood product manufacturers in China, and our investigation was restricted to plasma pools from two of these. Thus, the data obtained in this study does not reflect the nationwide status of B19V contamination of blood products in China and our results do not provide information about regional and temporal differences in contamination prevalence. Despite these limitations, our study represents the first investigation of B19V presence in Chinese blood products.

## Conclusion

B19V DNA was detected in Chinese plasma pools and plasma derivatives with some high titer. Due to plasma products are prepared from plasma pools and are administered to large numbers of patients, including some susceptible populations, such as pregnant women and immunodeficient patients. We propose to introduce B19V NAT into screening protocols for plasma donations and discard donation with high viramic concentration, with the aim of reducing the levels of parvovirus B19V in pools destined for fractionation, has the potential to improve blood products safety in China.

## Abbreviations

IVIG: Intravenous immunoglobulin; PCC: Prothrombin complex concentrate; S/D: Solvent/detergent; NAT: Nucleic Acid Testing.

## Competing interests

The authors declare that they have no competing interests.

## Authors’ contributions

WL designed, searched data and literature and gave a critical view of manuscript writing. ZW, LK, LC, ZY performed the experiments, collected and analyzed the data. All the authors’ read and approved the final manuscript.
